# Family Support and Diabetes: Patient’s Experiences From a Public Hospital in Peru

**DOI:** 10.1177/1049732318784906

**Published:** 2018-08-01

**Authors:** M. Amalia Pesantes, Adela Del Valle, Francisco Diez-Canseco, Antonio Bernabé-Ortiz, Jill Portocarrero, Antonio Trujillo, Pilar Cornejo, Katty Manrique, J. Jaime Miranda

**Affiliations:** 1 Universidad Peruana Cayetano Heredia, Lima, Peru; 2 University of North Carolina at Chapel Hill, Chapel Hill, North Carolina, USA; 3 London School of Hygiene and Tropical Medicine, Bloomsbury, London, United Kingdom; 4 Johns Hopkins University, Baltimore, Maryland, USA; 5 Hospital Nacional Arzobispo Loayza, Lima, Peru

**Keywords:** social support, diabetes, families, management, RCT, qualitative, Peru, in-depth interviews

## Abstract

Family support is crucial for managing chronic conditions but it is often overlooked when designing behavioral interventions in type 2 diabetes mellitus (T2DM). As part of the formative phase of a feasibility randomized control trial (RCT), we conducted 20 semistructured interviews with people with T2DM from Lima, Peru. Based on such results, we describe the support people with T2DM receive from their families and the role that such support has in their efforts to implement diabetes management practices. We learned that participants receive support from family members, but mostly from their spouses and children. Their relatives encourage them and motivate them to fight for their health, they also provide instrumental support by preparing healthy meals, reminding them to take medications, and sharing physical activity. Participants also reported controlling actions which were not always “well received.” Thus, any intervention supporting self-management practices need to work with key family members. We support the literature that suggests that interventions should target family members to ensure improved T2DM self-management practices.

## Introduction

In low-middle income countries (LMIC) like Peru, type 2 diabetes mellitus (T2DM) is a major challenge for the public health system and the people affected by it. In Latin America, only 3.6% of patients achieve the three American Diabetes Association recommended targets for blood pressure, low-density lipoprotein (LDL) cholesterol, and HbA1c (hemoglobin A1c) (Chan et al., 2009), which can cause diabetes complications and even early death. Self-management practices are crucial for T2DM management. Self-management practices are largely determined by the context in which they occur (Mayberry & Osborn, 2012) and family environments are crucial for helping or undermining self-management practices (Beanlands et al., 2005). Family support has a major impact on a patient’s ability to self-manage their chronic condition (Beanlands et al., 2005; Jennings, 1999). Friends and families can promote good health by influencing a person’s daily behavior, and the loss or reduction of such support can have negative health effects (Black, Maitland, Hilbers, & Orinuela, 2016).

Family environments and family relations are not homogeneous. They can be complex and so are the ways in which family members participate in promoting self-management practices (Tang, Brown, Funnell, & Anderson, 2008; Vongmany, Luckett, Lam, & Phillips, 2018; Wiebe, Helgeson, & Berg, 2016). There is still not a big body of literature that describes the best way in which family members should support T2DM self-management practices (Mayberry & Osborn, 2012; Rosland, Heisler, HwaJung, Silveira, & Piete, 2010) or the challenges families face to adapt to the needs of a relative living with T2DM at home (Beanlands et al., 2005). Friends and families are impacted by a patient’s health and his or her efforts to manage chronic condition such as T2DM (Black et al., 2016; Long, Jahnle, Richardson, Loewenstein, & Volpp, 2012).

According to Bennich (2017), T2DM affects family members differently either by improving family cohesion or causing psychological distress, but when and why this happens still needs further study. Families can be supportive, nonsupportive, or both. For instance, support can sometimes be expressed as control or the use of an authoritarian-style of supervision over the patient’s unhealthy behaviors, in hopes to encourage healthy behaviors and discourage negative ones (August, Rook, Stephens, & Franks, 2011; Newton-John, Ventura, Mosely, Browne, & Speight, 2017). The individual may view social control as positive or negative (Newton-John et al., 2017). Negative perceptions of social control can cause distress when people resent being directly monitored by others (Hughes & Gove, 1981).

This article describes the results of a qualitative study around the role of family members in providing support to their relatives with T2DM in Lima, Peru. We wanted to know who were the family members providing support, the shape of that support (buying medications, going to appointments with the patient, etc.) as well as the perception of such support in diabetes management from patients with T2DM.

## Method

### Design

This was a qualitative study that used a phenomenological approach to understand a wide range of topics related to the experiences of patients with T2DM in managing their condition. This study was part of the formative research of a feasibility randomized control trial (NCT02891382).

### Setting

This research was conducted at the Hospital Arzobispo Loayza, a third-level care facility located at the metropolitan center of Lima, Peru, the largest public hospital in the city. The hospital provides medical services to individuals of low socioeconomic status that include emergency medicine, geriatric services, and endocrinology. Additional nonmedical services such as nutrition, pharmacy, and social services are also available for patients at the hospital.

### Data Collection Tools

The semistructured interview guide was drafted by M.A.P. and F.D.C. The purpose of the interview guide was to collect information for the feasibility trial design as well as to have a better understanding of participants’ self-management efforts within the context of their experiences with T2DM and the social support they receive at home. The interview guide was tested on two participants by an interviewer, who conducted the remaining 18 interviews, and by a research assistant who was responsible for recruiting the participants. As both pilot interviews worked as planned, they were considered part of the pool of 20 interviews.

### Participant Recruitment

We had two strategies to recruit patients: asking endocrinologists to refer their patients to the interviewers, and convenience sampling. Recruitment was done by the interviewer and a research assistant at the hospital’s waiting room of the Endocrinology Department during a 2-week period in June 2016. The inclusion criteria required participants to have a diagnosis of T2DM for at least 1 year, be 18 years of age and over, and able to give consent to be interviewed. Patients were excluded if they had visible serious diabetes-related complications, such as blindness or amputated limbs. The chosen inclusion criteria for this formative study were set to parallel the eligibility criteria of the feasibility trial. The interviewer had previous experience in data collection for qualitative studies. She was given two 4-hour training sessions, where she received an explanation of the study, the aims of the formative research, and steps for obtaining informed consent from potential participants. Each interview question was reviewed carefully with the interviewer to ensure she was clear about the expected information we wanted to obtain. Patients who were invited to participate were told that we wanted to do a short anonymous interview to learn about their health status, their challenges around treatment adherence, and their opinion about a future study for patients with T2DM. To interview 20 patients, the interviewer and research assistant approached approximately 40 individuals. Patients who declined the interview did so because: (a) their medical appointment was about to start and they did not want to lose their turn, (b) they had to return to their work as soon as their appointment was over (this was a typical response received from men), (c) they had to return home to cook or take care of their children (usually women), or (d) they did not feel comfortable agreeing with the oral consent. Men were more difficult to recruit because they were a minority among the patients in the endocrinology waiting room, and because they had to return to work soon after their medical appointment. After each interview, participants were given 10 soles (~US$3.00) for transportation expenses, a pen, and a recipe book as a way to thank them for their time. These thank you gifts also incentivized other patients in the waiting area to participate.

### Interviews

All participants received the same questions, and the interviews averaged to a length of 50 minutes, with the shortest interview running at 19 minutes and the longest interview at 1 hour 15 minutes. Interviews took place at the hospital’s waiting area which afforded little privacy. However, except in two occasions, most interviews remained exclusively between the participant and interviewer. All interviews were audio recorded and some basic notes were taken during the interview, but these notes were used only to make further questions to the interviewee and not as additional information for understanding the results.

After the 20 interviews were conducted, the interviewer wrote a summary report of her initial impressions around the topics of (a) general experience with T2DM, (b) T2DM and social/family environment, (c) adherence to lifestyle changes, (d) attitudes and knowledge on obesity and its link to T2DM, and (e) opinions on participating in an intervention comprising monetary incentives. The summary report was useful in providing a first impression of the data collected, and to start thinking about the codes we might use for data analysis.

## Data Analysis

### Codebook Creation

All 20 audio recordings were transcribed verbatim, with only verbalized pauses being omitted from the transcripts. One of the authors (A.D.) reviewed the content of the transcripts and checked the quality by listening to the entire audio of two transcripts. Transcripts had inaudible parts of a few seconds long that did not affect the understanding of the content of their corresponding paragraph. Inaudible parts in the audio occurred either because of background noises or because certain patients, at times, had incomprehensible speech.

A.D. reviewed and read each transcript as they were received, and gave feedback to the transcribers regarding the quality of their prior audio. Transcripts were read and notes and memos were done related to the topics in the interview guide (see the appendix). A summary of each transcript was written at the end and an excel spreadsheet was used to track notes and observations made about each patient within the different interview questions. The excel spreadsheet was organized into four main categories derived from the interview guide as well as subcategories pertaining to their larger category as seen in Table 1. These categories help shape and identify a priori codes for all sections of the research questions as patterns became notable through the reading of the texts. For this article we looked specifically at data coded under category “changes and adherence to self-management behaviors.”

**Table 1.** Categories and Subcategories of Analysis Created From a Detailed Reading of Data.

Categories	Subcategories
1. Experiences living with type 2 diabetes	Diagnosis of T2DM
	Causes of T2DM
	Reaction to T2DM diagnosis
	Reaction to diagnosis from closest relatives
2. Changes and adherence to self-management behaviors	Dietary changes
	Types of support received for dietary changes
	Physical activity
	Types of support received for physical activity
	Medication adherence
	Types of support received for medication adherence
3. Attitudes and knowledge between obesity and T2DM	Thoughts on overweight/obesity impacting their health negatively
	Thoughts on overweight/obesity tied to T2DM
	Attitudes about their own weight
	Weight loss attempts
	Knowledge of HbA1c
4. Attitudes toward intervention using monetary incentives	Attitudes on receiving a prize for losing or maintaining weight for T2DM management
	Attitudes on losing 1 kg (2.2 lb) or maintaining healthy weight every 2 weeks
	Attitudes related to the monetary incentive, the amount proposed by participant and reasons
	Who is their chosen support person and why
	Thoughts on what type of sup[port their chosen person will bring to participant
	Barriers that limit participation in intervention

*Note.* T2DM = type 2 diabetes mellitus; HbA1c = hemoglobin A1c.

Codes related to behaviors around T2DM management primarily arose from the interview questions and the preliminary overview of the interviews. Some of these codes include “barriers to behavior changes,” “changes in diet,” “physical activity,” “medication adherence,” and “facilitators to behavior changes.” Codes outlining social support came from the interview questions, their corresponding answers (which went beyond the interview question), and from the literature, explaining different types of social support (a priori codes). Through further reading of interviews, a few interpretive codes arose such as “Exclusion” and “Exclusion-Purposeful.” These codes described feelings of alienation internalized by participants, or of being deliberately left out from social gatherings and family dinners because of dietary restrictions caused by their T2DM condition.

A first draft of the codebook was created and shared with M.A.P. and F.D.C. who provided feedback. A second and a subsequent third draft were created with codes shifting toward a generalizable application to the different themes and subthemes within each transcript. Once the codebook was finalized, it was uploaded onto Atlas.ti.7, along with the 20 transcripts. For quality control of the coding process, A.D. coded one transcript using all the codes created to ensure reliability and understanding. Minor revisions to the codebook were made once the coded transcript was reviewed. Afterward, all remaining transcripts were coded on self-management behaviors (medication adherence, food consumption and physical activity), support received related to these self-management behaviors, patients’ perspective around participation in a mixed-economics incentive intervention, and weight loss attempts. These codes were subsequently exported into MS Word documents from Atlas.ti.7 for cleaning and to see which codes interacted with one another. A total of 29 codes were pulled and analyzed (see Table 2).

**Table 2.** Codes Used to Explore Social Support.

Code	Definition
Acute hospitalization	Apply this code when patient mentions being hospitalized as a direct result of medical conditions caused by mismanaged type 2 diabetes care, and was hospitalized longer than a day.
Barriers to behavior changes	Apply this code to describe barriers or situations patient faces that make it difficult to initiate and sustain the self-management of behaviors that improve type 2 diabetes condition.
Behavior change sustainability	Apply this code to highlight the spectrum of sustainability related to behavior changes participant has attempted to self-manage their type 2 diabetes care and/or weight control related to areas in their diet, physical activity levels or medication adherence.
Changes in diet	Apply this code when participant describes dietary changes related to type 2 diabetes self-management
Chosen support person	Apply this code when a participant describes reasons that increase the difficulty of them participating in the intervention or prevent them from participating at all
Cost burden of care	Apply this code when patient mentions difficulties in managing care because of increasing cost of diabetes management and comorbidities.
Current attitudes toward T2DM	Apply this code to highlight how a patient currently describes their feelings and attitudes around being a person with type 2 diabetes
Diabetes complication	Apply this code when patient mentions developing a comorbidity or lasting medical condition related to diabetes as a result of mismanagement of type 2 diabetes care.
Emotional support	Apply this code when someone in the patient’s social network provides emotional support that improves the patient’s ability for diabetes self-management and/or weight control.
Exclusion	Apply this code to situations, examples, and events where patient claims feelings and attitudes of exclusion or experiences isolation from people, behaviors or situations because of their type 2 diabetes, and does not fit within child code below.
Exclusion-purposeful	Apply this code when patient purposely separates self from activities or behaviors done by others, to better manage their care.
Facilitators to behavior change	Apply this code to describe facilitators that help sustain self-management of behavior practices that improve type 2 diabetes management and/or weight control.
Family assimilation	Apply this code to highlight the varying degrees to which patient’s closest and secondary support adopt the lifestyle changes that help patient improve type 2 diabetes management and/or weight control.
Instrumental support	Apply this code when there are examples of people in patient’s social network providing tangible and observable support that helps directly or indirectly improve patient’s diabetes self-management and/or weight control, and does not fit with child codes below.
Instrumental support-financial	Apply this code when those in patient’s social network directly provide financial assistance to help patient directly or indirectly manage their type 2 diabetes care and/or weight control.
Medication adherence	Apply this code when participant describes medication adherence related to type 2 diabetes self-management.
Motivation for intervention participation	Apply this code to describe the reasons participants say for themselves or in general for participating in the mixed-economics incentive intervention after it has been explained to them.
Noninvolvement	Apply this code when participant mentions certain members in their social network or in general not involved in their type 2 diabetes self-care and/or weight control.
Other support	Apply this code when patient mentions perceiving or receiving other support related directly or indirectly to type 2 diabetes self-management that does not with other parent social support codes.
Past attitudes toward T2DM	Apply this code to highlight how a patient describes their feelings around being a person with type 2 diabetes shortly after receiving diagnosis.
Physical activity	Apply this code when participant describes physical activity related to type 2 diabetes self-management.
Sabotaging support	Apply this code when social network intentionally behaves in a way that conflicts or makes participant’s diabetes self-care efforts and/or weight loss more difficult.
Social control-positive	Apply this code when patient mentions that the direct control over aspects of self-management by person within social network has been helpful in managing their own care.
Social control-negative	Apply this code when patient mentions or suggests that the direct control over aspects of self-management by person within social network has not been helpful in managing their own care.

*Note.* T2DM = type 2 diabetes mellitus.

### Interpretation of Data

We used a deductive analysis at the beginning since the major themes derived from the a priori established topics regarding patient’s social support network. Afterward, we identified that people’s descriptions matched well with concepts derived from social support theories and we organized our data along such topics. We used a thematic content analysis approach aiming to capture participants accounts (Green & Thorogood, 2014), but we also borrowed some insights from phenomenological approaches as we wanted a closer understanding of people’s experiences and actions as patients of a public hospital with T2DM (Abolghasemi & Sedaghat, 2014; Starks & Trinidad, 2007).

### Ethics

The study was approved by the institutional review board at Universidad Peruana Cayetano Heredia and at the Hospital Nacional Arzobispo Loayza. All participants provided oral informed consent before beginning the interview.

## Results

### Demographics of Study Participants

We interviewed 20 participants, out of which 15 were female (see Table 3). The average age of participants was 55 years (range: 43 to 69 years). Participants had an average of 12 years living with T2DM since first diagnosis, with individuals varying widely in time since diagnosis from 1 year and 8 months to 25 years. Eighteen participants mentioned their living situation (if they lived alone or with other family members). Six reported living with a spouse/partner only, four reported living a spouse/partner and at least one underage child, and two reported living with at least one adult child (in both cases it was a son). One participant reported living with their parent (mother), one reported living with an extended family member (an uncle), and one participant lived with an underage grandchild. Two participants reported living alone.

**Table 3.** Participants’ Characteristics.

Total Participants Interviewed	20
Female participants	15
Male participants	5
Participant age (*n* = 20)	
General average	55 years
Female average	57 years
Male average	49 years
Average weight (kg) of participants	77 kg
Self-reported cohabitation (*n* = 18)	
Living with an underage grandchild	1
Living with at least one adult offspring	3
Living with a spouse/partner only	6
Living with a spouse/partner and underage child	4
Living with extended family	1
Living with a parent	1
Living alone	2
Self-reported medication use (*n* = 20)	
Metformin only	5
Insulin only	4
Insulin and dialysis	1
Metformin and Insulin	5
Insulin and Glimepride	1
Metformin and Glimepride	2
Metformin and Glyburide	2

Participants mentioned that the majority of help received for T2DM self-management in their household came from spouses and grown-up children. Participants also mentioned that family who did not live in their household (nephews, siblings and other adult children), also provided support. In the following sections we will describe in detail the type of support that was given and the perception of such help from the patients.

### Encouragement and Care

Participants expressed having feelings of distress and hopelessness stemming from the challenges of self-management practices, their diabetes prognosis and/or from emergency care events related to their illness. In these situations, family members played an important role in uplifting the patient’s spirits, showing empathy and trying to alleviate their distress. For example, the daughters of one participant, upon learning that their mother had T2DM, stressed the participant’s good qualities and capacity to face difficulties (“(mom) you are a warrior”). Two other participants told us that both their spouses and children offered words of encouragement and empathy, while stating their unwavering support. As participants recall,

[When I learned I had diabetes] I was worried that I would die, and my father would tell me that I was not going to die. “Look at me, I’ve had diabetes for so long and I haven’t died yet.” So now I have my psychologist, my children, my husband telling me to stay calm. (Female, age 52)

[My son] told me, “mom, don’t feel sorrow, everyone in the world is a diabetic, you can live a normal life, I will help you.” It’s because of him that I am alive today, miss. (Female, age 58)

Besides receiving emotional support, we also encountered participants who received financial assistance from their children. The money was mostly to pay for bills and medications:

. . . when I return from the hospital my daughters ask me for the [medication’s] bill and ask me what I have [been able to get] and what not. And my eldest daughter quickly gathers her brothers to buy me all of them. For instance, last month there wasn’t insulin and they had to buy it, and it’s expensive. (Female, age 53)

Financial support was complemented with other caring activities such as encouraging them to rest, suggesting them not to work, and to take things easy (“don’t worry, relax”). For instance, one participant explained that his relatives pooled money together and paid for him to go on a vacation as a way to cheer him up from the sadness he felt from being recently diagnosed with T2DM,

My family has never let me go without [help]. Since I like to travel, . . . at home they told me, “Fatty you are going to Huancayo”^1^ and I said: “No,” but they said “Go, we will pay for your ticket.” So with that esteem, with that support from the family, I kept going. That has helped me get better. (Male, age 50)

In addition, family members would visit participants regularly at their home or the hospital (usually for complications), and also extended open-door policies to participants (“My daughter would tell me, ‘dad, I love you, come over anytime to my house.’”).

Compassion, encouragement and solidarity with participants in combination with financial assistance and companionship, were common expressions of support by family members.

### Walking Side by Side

Besides emotional and financial support, lifestyles changes were backed by engaging with and accompanying their relatives in the new behaviors. Participants shared the way in which family members engaged in their care. Family members learned about T2DM management and made efforts to incorporate into the family life, aspects of the lifestyle changes participants had to undergo. One participant described how his wife was always by his side during medical appointments, and that she attended educational sessions to learn about diet restrictions for people with T2DM. Another participant explained how her daughters encouraged her to include them in her new dietary habits,

They tell me, “But, mom, why do you cook spaghetti? Cook other things, mom.” I tell them that “just because I am sick, it doesn’t mean that I have to give you the same things I eat.” They tell me, “But you would be taking care of us as well.” (Female, age 53)

Five participants reported having family members accompanying them on walks as part of their efforts to engage in recommended physical activity. This, as mentioned before, goes side by side expressions of love and care:

My son tells me, “mom, are you going to go on a walk? Let’s go, get the dog, all three of us will go.” We go around the area several times. I walk until Angamos Avenue, Paseo de la Republica Avenue, and then return home. We do that at night, with this heat we don’t walk, if its night we go out for walks when he returns from the university, and he tells me “Let’s go mom. Eat first, I’ll eat later.” (Female, age 52)

We also found family members who lend participants a stationary bike or set alarm reminders for participant’s morning walks. Four participants mentioned having a family member in their households that helped them with insulin shots. Other participants mentioned how family members would bring them a glass of water to take with their medications, had participants’ medications on hand, or ensured participants did not miss their medical appointments.

### Vigilance and Control

We also learned that just as there were supportive practices there were some actions that participants considered acts of vigilance or control over their health behaviors. Depending on the circumstance, these types of actions could be perceived as helpful or not helpful for participants’ T2DM self-management practices.

For instance, one participant shared how it was her son’s scolding and warnings to instill fear about the consequences of poorly-managed diabetes that motivated her to be mindful of her condition.

My son (who is) thirty years old, takes care of me a lot, he is the one that takes care of me the most. Otherwise I would have died. About three years ago (he told me), “mom, take notice (of your health), please.” He would take care of me and I would go out and eat rotisserie chicken, fries . . . Until one day he shook me, “you are going to die, go blind, be placed on dialysis. Your legs, arms and fingers will get amputated. Take notice!” Yeah, that scared me, yeah. (Female, age 58)

In addition to advice or scolding, family members also provided direct assistance in everyday self-management activities. Several participants reported having relatives constantly ensuring they remembered things:

[My family] tells me, “take your crutches, make sure you are taking your ticket to the appointment, make sure you are taking your syringes, don’t forget anything.” They are helping me remember things. Like I say, I have support. Other sisters who live further away come and visit me. They ask me, “do you have money for your transportation? Here you go.” (Male, age 50)

This example highlights direct assistance for consistent T2DM self-management that still provides participants with a sense of autonomy in their own T2DM care. However, participants also gave examples of when family members’ engagement with their T2DM care would take on a directive and controlling approach, particularly in the areas of food intake and physical activity. Family members, usually spouses and children, would tell participants to be mindful about what they consume (“you can eat a small fruit, not a huge one”), while also preparing healthy foods such as salads or low-sugar lemonade. Participants sometimes appreciated these behaviors since they felt cared-for. However, in other instances such actions were interpreted as restrictive. For example, to further guarantee healthy food consumption, family members kept unhealthy foods, such as sodas, cakes or sweets, away from participants.

. . . in my house no one allowed me to drink soda. Just water, lemonade without sugar, just that. That is why I stopped drinking soda, because before that, I was a soda fiend, drinking two or three liters daily. But I don’t drink soda anymore. (Male, age 47)

[My children tell me] “Now you have to take care of yourself, you can’t eat anything that is sweet; you have to forget about cake.” (Female, age 52)

Directive behaviors toward physical activity were also reported. Participants mentioned family members telling them to go out and exercise, and one participant mentioned how her son enrolled her in dance lessons to increase her level of physical activity.

. . . [my son], he told me, “mom, do you want to be lazy during the evenings? Just lying there watching TV, living a sedentary life? Don’t you want to go dancing or for a walk?” “I don’t know,” I told him, “I like dancing. Marinera, Salsa, anything.” “Ok, mom, I enrolled you in Marinera,” and so I go to my dance class once a week. (Female, age 58)

Although these examples showcase moments when participants did not mind the authoritative intervention, and may regard it as helpful in promoting their T2DM care, not all controlling behaviors were welcomed or thought of as helpful. During the interviews, participants described how family members’ controlling actions or verbal chastising caused distress,

(I feel) that they are controlling me, that they are too much into what I eat: “mom don’t eat too much”; “mom, watch out for this”; “mom, not that”; “mom, you need to cook this,” “mom, you are eating again?” It gets tiring, no? . . . Well I tell them: “do you want me to starve?” (Female, age 56).

Another participant resented her children constantly telling her to walk, climb the stairs and be active in a manner that she perceived as harsh and authoritarian “do this, do that.” Some participants expressed experiencing physical pain as a result of diabetes complications which prevented their mobility and endurance. These participants were upset that their families could not understand how their physical condition drastically limited the types of physical activities they could perform and their frequency. As a result, participants did not find it helpful when family members asked them to exercise.

And I walk if I need to walk . . . But (my knee hurts) that’s why I avoid walking. That’s why I sometimes tell my sisters: “if you knew how my knee hurts, you would not tell me: ‘sister you have to walk’” because it hurts too much. (Female, age 62)

Just as some participants appreciate family members’ controlling behaviors, some participants resented the constant supervision and reminders to take care of themselves. These reminders were perceived as scolding or nagging behaviors that did not offer alternatives or solutions to challenges and obstacles participants experience in T2DM self-management,

My daughters are the ones that worry. “Your diet, you have to take care of yourself.” They always watch out for me. At times, it becomes bothersome, like it tires you, it makes you uncomfortable.,you know. I tell them “when I die, I’m taking my coffee with me, nothing else.” That’s what I tell them. (Female, Age 56)

Subsequently, it appears that nagging behaviors from family members was perceived by participants as a sign that family members are indifferent to the participant’s struggle to maintain their T2DM self-management, as expressed by one participant,

They don’t care about me, or think that I am only there to attend to their needs and nothing more. They only tell me to make sure I’m not eating sugar or too many carbohydrates, nothing else. (Female, age 58)

### Social Exclusion and Isolation

Besides the supportive and/or intrusive practices described, participants also shared how sometimes they felt marginalized by their families. Not adapting their eating habits to match those of the person with diabetes was a practice that participants particularly resented as they considered their relatives were not making an effort to take into account the participant’s dietary needs and restrictions. Some participants described how family members’ food consumption habits undermined efforts to, or created additional challenges in, T2DM self-management. One participant described having to cook separate food items for herself in addition to what she cooks for her children.

Look, I have learned to take care of myself now because I do not want to get sicker. I learned to take care of myself, and I put in a lot of effort because sometimes I eat dinner with my children and I eat something different. They tell me “mom, I want to eat rice with chicken” and I have to eat something different. (Female, age 52)

Another participant described how visiting his parents also means compromising his dietary regiment for T2DM self-management,

For example, when I go to my parent’s house, they know that I am like this [person with diabetes]. The only thing they make me is a small salad, but then I have to eat whatever else they cook. (Male, age 55)

These differences between the diet of family members and participants reminded participants during mealtimes about what they cannot consume anymore, and how their condition separates them from their family. It also gave participants the impression that their family does not understand, or care enough, about the severity of their condition. This made some participants feel excluded from their family networks especially in venues where food is a key element (birthdays, mother’s day, Christmas). At least seven participants expressed having feelings of isolation and exclusion when they socialized with friends and family, particularly because of the restrictive diets participants need to follow for optimal T2DM self-management. Furthermore, several participants expressed feelings of sadness at not being able to eat the food items, such as sweets, they enjoyed prior to their diagnosis or, in some cases, after experiencing a hospitalization occurrence after diagnosis. Participants mentioned their fears of eyesight loss or foot amputation due to poorly-managed T2DM care.

Yes, I suffer because I see how they eat and I can’t eat the same amount I ate before. I suffer; sometimes at night, I drink my anise tea and eat a piece of toast and go to my bedroom and cry, thinking, “why can’t I eat the same?” I say “God help me.” My daughter asks me if I want to eat something, that she’ll give me a little, and I tell her no, no . . . because I have to lose weight. (Female, age 53)

During the interviews, some participants shared that, in response to their families not adopting similar eating habits, they preferred to avoid sharing meal time with their families as a way to prevent the possibilities of sabotaging their diet-related self-management efforts.

Sometimes, when I see my children eat, I avoid sitting at the table. My son-in-law asks me why I don’t sit at the table, I tell him I need to finish something and, later, I will eat alone. I see all those plates and I want to eat the same, which is why I need to avoid being tempted. (Female, age 53)

This self-imposed isolation extended, for some participants, into also avoiding social gatherings because of fears that the presence of unhealthy foods, and the direct or indirect pressure of family and friends consuming these foods, would tempt them into eating things that may worsen their T2DM condition.

. . . at times you can’t eat what others normally eat, no? You refrain from going to a gathering because you can’t drink beer, you are there with your glass of water. You refrain yourself from many things, many foods you can no longer eat. You have to leave all of that because of your illness. (Male, age 47)

When I go to a get together, I cannot eat this or that. I cannot try a single snack, so it’s better for me not to go. Sometimes they prepare goat and that’s all grease you’re eating, and, so, I will like, “no thanks,” which is why I avoid such gatherings. “No,” I tell them, “you go on.” They tell me, “mom, you are depriving yourself too much, go and live your life normally. Just watch what you eat.” . . . Seeing those big-eaters will only tempt me. (Female, age 52)

Finally, two participants mentioned that their diabetes makes them feel tired and sleepy all the time which is something they see as a negative side-effect of their diabetes that impacts their social life.

## Discussion

Through our results, we can see how family members participate in participants’ T2DM self-management and how they engage in behaviors that are supportive, controlling, or that can undermine efforts. Supportive behaviors included the provision of emotional support, such as empathy and alleviation of diabetes-related distress, and the provision of instrumental support such as paying for medications and helping participants apply insulin. Controlling behaviors around T2DM self-management translated into vigilance over certain lifestyles and into nagging participants to watch what they eat and to be physically active. The reaction to controlling behaviors varied. Some participants interpreted controlling actions from family members as expressions of care and found them helpful. Others viewed controlling behaviors as unhelpful and annoying. Several participants also lamented that their relatives were indifferent to their needs and often, offered them (or ate) unhealthy foods in front of them. As a result, several participants avoided attending family social events, where “forbidden” foods will be served.

Our study adds to the growing literature around family supportive and nonsupportive behaviors for T2DM self-management (Bennich et al., 2017; Hu, Wallace, McCoy, & Amirehsani, 2014; King et al., 2010; Rosland, 2009; Rosland et al., 2010; Vongmany et al., 2018). We documented instances in which the help offered by family members was not well taken by those with T2DM, especially around dietary restrictions. Similar to other studies, we found that family members could both be supportive and obstructive (Knutsen et al., 2017; Schwingen et al., 2015). According to Bennich (2017) those family members who incorporate aspects of T2DM self-management into their everyday routines are also the ones that engage in other supportive practical actions and emotional behaviors. However, Mayberry, Harper, and Osborn (2016) found that 64% of their participants reported that they experienced obstructive and supportive behaviors around T2DM management from the same person. Our data does not allow us to determine if those who formed positive care partnerships with participants were the same that at times, made it difficult to put into practice self-management behaviors. However, we did learn that oftentimes being watchful over the participants’ T2DM condition could either be interpreted as care or end up undermining T2DM self-management efforts. These findings are in line with a systematic review on family behaviors that impact T2DM self-management activities (Vongmany et al., 2018).

Our study contributes to understanding the complexity of family support as something that cannot be only seen as positive or negative, but rather that has to be understood in context and through the lens of the patient. As other authors have mentioned, patients with chronic illness do not always experience family involvement positively (Fritz, 2015; Rosland et al., 2010). Studies have shown that when family members try to support their relatives with T2DM, they can feel criticized, nagged, or even guilty (Carter-Edwars, Skelly, Cagle, & Appel, 2004; Pitaloka & Hsieh, 2015; Trief, Sandberg, & Greenberg, 2003). In some cases, this type of “support” can led to negatively impact patients’ outcomes (Vongmany et al., 2018).

There is more yet to learn regarding family support and going beyond those who live in the same household. For example, in our study, when we asked participants who in their household helps them with different aspects of T2DM self-management, most mentioned that a spouse/partner and adult children acted as the primary providers of assistance and involvement in areas of T2DM self-management, followed by adult siblings and other family members. But we learned that there were other relatives living outside of their household involved in T2DM care. Our participants’ description of which family members are involved, living within or outside the household, and their household make-up align with two other studies whose participants reported similar family support and household conditions (Hu et al., 2014; Mayberry et al., 2016).

### Public Health Relevance

It is becoming increasingly clear that the social environment of persons living with T2DM and their interactions with those in their personal networks influence their T2DM self-management. The extent to which members within networks can adequately respond to the needs of someone with T2DM (e.g., monitoring, medication, physical activity, and diet management) depends on members’ knowledge and competence around T2DM care (Vassilev, Rogers, Kennedy, & Koetsenruijter, 2014). Family networks are important to look at because most T2DM self-management practices occur in the home (Mayberry & Osborn, 2012). A recent systematic review found, despite the evidence of the importance of social support and social networks for chronic disease management, very few interventions focus their work in such networks, emphasizing the individual (Spencer-Bonilla et al., 2017). The concept of *collective efficacy* can help strengthen and amplify the provision of supportive behaviors from family members toward T2DM management and also promote active involvement in T2DM care. Collective efficacy is “a shared perception and capacity to successfully perform and behave through shared effort, beliefs, influence, perseverance, and objectives” (Vassilev et al., 2014). Focusing on the collective efficacy of the family network around T2DM management can improve family member(s)’s ability to perform supportive behaviors and share the burden of care. Collective efficacy for improved T2DM management may involve promoting shared goals toward glycemic control, shared knowledge of the disease, and integration of promotive lifestyle changes within the network. The latter can be helpful for persons living with T2DM since they do not want to feel singled out as the “diabetic” (Mayberry et al., 2016).

Another potentially important aspect for leveraging family networks and improving T2DM involvement is by helping family members understand their role in T2DM care. Our study points to the importance of helping family members recognize which types of controlling behaviors are helpful and not helpful in management, and to understand situations in which it is appropriate to exert a degree of social control for T2DM management. Members in networks can become overly concern and over-controlling in response to a person’s T2DM diagnosis (Vassilev et al., 2014). Controlling behaviors can be beneficial in circumstance where persons living with T2DM has trouble initiating or sustaining T2DM self-management changes (Mayberry et al., 2016). However, excessive control from family members can negatively influence T2DM self-care or cause person to avoid seeking assistance from their network to retain autonomy (Mayberry & Osborn, 2012; Vassilev et al., 2014). When and why social control is perceived as positive or as negative by the receiver has yet to be studied more in depth.

Overall, interventions and programs that promote family support and involvement T2DM care can think about how to help family members navigate this chronic conditions by helping members develop skills required to undertake more complex behaviors for management that go beyond commands and directive actions. Focusing on the collective efficacy of the family network can be used to promoted engagement in more supportive behaviors because it has members involved in shared goals for better chronic disease management and also allow for integration of habits such as physical activity and diet in the network to support individual living with T2DM. Moreover, family members may be better able to engage in behaviors that promote T2DM management by understanding their role in care. Furthermore, they will also understand that self-management behaviors are negotiated with the person living with T2DM and that controlling behaviors the network may engage in can have positive impacts, when promoted at the appropriate time and place. These shifts combined with training family members can help move more behaviors from controlling to supportive or at the very least have controlling behaviors accepted and appreciated by participants.

### Limitations

Our study focused on learning the different ways in which family members supported T2DM self-management practices. Our collected data primarily reviews supportive and controlling behaviors as described by our participants and we did not collect data about participatory behaviors for T2DM management from the perspective of those family members. As a result, we could not get a full scope of the interaction between received and perceived support from both participant and their family member(s). We did not observe the participants in the context of their home environment or other places they frequent where they receive some varying forms of social support or control from those in their networks.

However, our questions regarding family support were directed toward identifying one specific provider (for the trial), when, as we found out through our interviews, there is a larger family network of care behind the support received by participants. We did not explore the full web of people who give varying degrees of support and/or control outside of the household.

Out of the 20 participants only five were men, so we lack more detailed information about men’s experiences with social support for self-management. Furthermore, our study could not capture “invisible support” which refers to those instances in which support is offered by it is not perceived by the patient and that only the relative could mention.

Future studies can look more at teasing out the complexity of social control that family members exert and help persons living with T2DM. It couls also look at how family members negotiate their roles in management such that family can know when it is appropriate to engage in controlling behaviors and the importance of respecting a person’s need for self-determination in their T2DM management.

Furthermore, information should be collected both from patients and family members to understand both sides of support for chronic care.

## Conclusion

Individuals with chronic conditions receive help to manage such condition from family members, mainly spouses and children. The forms of assistance can vary from encouraging self-management practices to social control expressed as restricting the patients’ actions that are contrary to doctor’s prescriptions. Our study shows that social support for T2DM management is mixed, sometimes those in their networks provide help, but at times, they undermine their relative’s effort. This is consistent with literature on the issue of social support and chronic conditions.

Social support is a heterogeneous concept and, as the literature says, there are multiple pathways by which social support can influence mental and physical health. Social support plays a role in preventing stress, it buffers stress factors experienced by the patient and it enhances quality of life. Our data mostly describes the latter role: enhancement in the quality of life among patients with T2DM. However, it is also important to think about when social support and/or being part of a social network is more relevant for a patient’s coping mechanisms. As a recent article states, social networks might be more relevant when the health system is weak or is not accessible for everybody (Spencer-Bonilla et al., 2017).

Participants received mostly emotional support from their family members who expressed their concern toward their condition and expressions of encouragement and care. Usually such emotional support was seconded by instrumental support to enable patients to improve their diet, follow their treatment and increase their physical activity. One useful concept to understand the interpretation of the support received in *familismo*, which has been developed to characterize the feelings of mutual obligation and respect that occurs within Latino families. Our research shows that social support has to be understood within its particular cultural context which should be part of the framework of any intervention that aims at using existing social support to improve diabetes management.

Finally, our study is an example of the relevance of qualitative research to better understand the context of patients before launching an randomized control trial (RCT). The study on family support was meant to fine-tune the activities of a feasibility RCT that aimed at improving self-management practices of T2DM patients using individual and mixed economic incentives. Mixed incentives are incentives that are given to both the participant and a person who would provide support to achieve the proposed goal. The qualitative study provided ideas about who could be those supportive partners and the type of information about diabetes management they needed to aid the patient.

However, it is important to mention that qualitative data analysis takes time since you need to familiarize yourself with the data and be thorough in your interpretation, and the process of designing the delivery of an RCT need input fast. So, qualitative formative research in the context of an RCT needs to take into account that it needs to be able to provide specific suggestions based on a first analysis of the data in a timely fashion.

## Supplemental Material

Online_appendix – Supplemental material for Family Support and Diabetes: Patient’s Experiences From a Public Hospital in Peru Click here for additional data file. Supplemental material, Online_appendix for Family Support and Diabetes: Patient’s Experiences From a Public Hospital in Peru by M. Amalia Pesantes, Adela Del Valle, Francisco Diez-Canseco, Antonio Bernabé-Ortiz, Jill Portocarrero, Antonio Trujillo, Pilar Cornejo, Katty Manrique and J. Jaime Miranda in Qualitative Health Research

## Notes

1. City in the Andean region.
