# Supplementary material for: Family Support and Diabetes: Patient’s Experiences From a Public Hospital in Peru
Source: Qual Health Res. 2018 Aug 1;28(12):1871–82. doi: 10.1177/1049732318784906 (PMC6346298; doi:10.1177/1049732318784906)
Supplement: Online_appendix – Supplemental material for Family Support and Diabetes: Patient’s Experiences From a Public Hospital in Peru [file Online_appendix.pdf]

Interview Guide

General Experience with Diabetes: General Consequences
Tell me, how did you find out that you had diabetes? *Explore the process.*
- Why do you think you got diabetes?
- Do you know what causes or produces diabetes? *Explore.*
- At the moment, how do you feel about being a person with diabetes? Why?
- Who of those close to you- for example, your partner, family or friends- know that you have diabetes?

Adhering to and Making Lifestyle Changes
Normally, it is recommended for people living with diabetes to take medicine, change their diet, and be physically active (or exercise). Which of these things has your doctor recommended?

I would like to know how you are doing in these three areas:

Pharmaceutical Treatment
What types of medications have you been prescribed for diabetes?
Do you take them exactly as you've been told? *If not*: Why?
Do you have difficulties in following your treatment? What kind?
How do you obtain your medications? (Example: obtain them for free, pay for medications, etc.)
Is there someone at home that provides support so that you can follow you diabetes treatment? How do they help you?
If no one helps you, why do you think that is?

Dietary Changes
Has your food intake changed since your diabetes diagnosis?
What have been the most important changes?
How easy or difficult has this been? Why?
Is there someone at home that provides supports so that you can make these changes in your food intake? How do they help you?
If no one helps you, why do you think that is?

Physical Activity
Has your level of physical activity changed since your diabetes diagnosis?
In what way?
How easy or difficult has this been? Why?
Is there someone at home that provides support so that you can increase your level of physical activity? How do they help you?
If no one helps you, why do you think that is?

Is there something else in which your family supports you in so that you may manage your diabetes that was not mentioned?

Knowledge and Attitudes about Obesity and Diabetes:
Do you know how much you weigh, approximately?

How do you feel about your current weight: are you satisfied or not satisfied? Why?
Would you like to lose, gain or maintain your weight? Why?
Have you, at some point, tried losing weight?
When?
Why?
What did you do? How did it go?
What problems did you have?
What helped you? Who helped you?

If not yet mentioned, explore:
Do you know if being overweight or obese, in other words being a bit fat (gordito), is good or bad for your health? Why?
Do you know if there is a relationship between diabetes and weight? Explore

Do you know what is glycosylated hemoglobin?
Do you measure your glycosylated hemoglobin?
How do you measure it? How often?
If they don't do it: why not?
What is a bad glycosylated hemoglobin value?

Opinions about the intervention:

Introduction to the intervention proposal:
"Image that we invite you to enroll in a health program in which we give you a price, in soles, if you manage to lose weight as a way to control your diabetes. In this program, you will come to the hospital every two weeks to meet with a nurse who will evaluate your weight. If you manage to lose weight (1 kg every two weeks) you will receive a monetary price. The intervention would last nine months."

What do you think of the idea of receiving a prize for losing weight for diabetes control?
What is good about this idea?
What is bad about this idea?
Would you be willing to participate?
Why would you do it?
Why would you not do it? *Or*, what doubts would you have?
What do you think about losing one kilo every two weeks?
Is it possible for someone like you to do it? Why?
Would it be possible to do it within a few months? Why?
What monetary amount would motivate you to change your habits and lose one kilo every two weeks? Why do you think this is an adequate amount?

Like we said, it is necessary to incorporate changes in your diet and physical activity.
Who would be able to support you?
Why would you chose this person?
How do you think this person could help you?
Would this person be willing to help you?

→ *If they say no:* Would the person be willing if you were to give part of the monetary prize with them?

The idea would be to come to the hospital every two weeks, for half an hour, to be weighed and speak with a nurse.

What do you think about coming every two weeks and staying for half an hour? Would you be able to do this? *Explore barriers.*

Would your support person be able to accompany you every two weeks? *Explore barriers.*
